# More evolvable bacteriophages better suppress their host

**DOI:** 10.1111/eva.13742

**Published:** 2024-07-04

**Authors:** Elijah K. Horwitz, Hannah M. Strobel, Jason Haiso, Justin R. Meyer

**Affiliations:** ^1^ Department of Ecology, Behavior and Evolution University of California San Diego La Jolla California USA

**Keywords:** antimicrobial resistance, bacteriophage, evolvability, phage therapy

## Abstract

The number of multidrug‐resistant strains of bacteria is increasing rapidly, while the number of new antibiotic discoveries has stagnated. This trend has caused a surge in interest in bacteriophages as anti‐bacterial therapeutics, in part because there is near limitless diversity of phages to harness. While this diversity provides an opportunity, it also creates the dilemma of having to decide which criteria to use to select phages. Here we test whether a phage's ability to coevolve with its host (evolvability) should be considered and how this property compares to two previously proposed criteria: fast reproduction and thermostability. To do this, we compared the suppressiveness of three phages that vary by a single amino acid yet differ in these traits such that each strain maximized two of three characteristics. Our studies revealed that both evolvability and reproductive rate are independently important. The phage most able to suppress bacterial populations was the strain with high evolvability and reproductive rate, yet this phage was unstable. Phages varied due to differences in the types of resistance evolved against them and their ability to counteract resistance. When conditions were shifted to exaggerate the importance of thermostability, one of the stable phages was most suppressive in the short‐term, but not over the long‐term. Our results demonstrate the utility of biological therapeutics' capacities to evolve and adjust in action to resolve complications like resistance evolution. Furthermore, evolvability is a property that can be engineered into phage therapeutics to enhance their effectiveness.

## 1 INTRODUCTION

The growing threat of antibiotic resistant bacteria has generated interest in the use of alternative therapeutics, such as bacteriophage (Gordillo & Barr, 2019; Kutter et al., 2010). Bacteriophages are natural enemies of bacteria and have been successfully used to treat multidrug resistant bacterial infections (Broncano‐Lavado et al., 2021; Chan et al., 2016; UC San Diego School of Medicine Center for Innovative Phage Applications and Therapeutics, 2024). Phages are ubiquitous in nearly every environment and are more genetically diverse than any other taxonomic group. This means that there are nearly endless varieties to harness (Batinovic et al., 2019; Jurczak‐Kurek et al., 2016; Zablocki et al., 2015). In contrast, antibiotic drugs are far fewer and new discoveries are rare (Brown & Wright, 2016; Donadio et al., 2010). To understand phage's enormous potential, take for example a single human pathogen, Actinobacter, for which researchers have collected a library of more than 20,000 bacteriophages that infect just this single phylum of bacteria (Russell & Hatfull, 2017). This incredible diversity is beneficial for providing practitioners with many options for treatment, but it also necessitates the development of criteria for selecting the best phage from among numerous options.

Currently, researchers select phages by first evaluating which can infect a pathogen of interest (Hyman, 2019). If multiple candidate phages are identified, then additional phage traits might be considered. Current literature recommends stability as one such trait (Casey et al., 2018; Hejnowicz et al., 2014), and mathematical models have been used to argue that stable phages will be more suppressive than unstable phages (Bull et al., 2019). There is also interest in deliberately enhancing stability in therapeutic phages via directed evolution (Favor et al., 2020). Another trait that is thought to be desirable in therapeutic phages is a high reproductive rate (Casey et al., 2018). The reasoning is intuitive: phages able to rapidly generate a large population should be more effective at arresting bacterial growth (Bull et al., 2019). Despite the appeal of both stability and reproductive rate, the importance of these traits in determining bacterial suppression has not been demonstrated empirically.

A third trait that has been relatively overlooked as a criterion for therapeutic phages is evolvability (Bono et al., 2021), defined as the capacity for adaptive evolution. The reason antibiotics and even phage treatments become obsolete is that bacteria evolve resistance (Blair et al., 2015; Labrie et al., 2010). However, unlike antibiotics, phage have an endogenous algorithm—evolution by natural selection—to overcome resistance by gaining counter defenses (Borin et al., 2021; Bull et al., 2019). Ideally, phage therapeutics would be able to evolve counter measures to resistance during treatment without any human intervention. Phages have been shown to vary in their capacity to evolve counter‐defenses, so favoring evolvable phages during therapeutic selection might yield a more powerful treatment (Casey et al., 2018). One mechanism by which bacteria evolve resistance to phage is by mutating, deleting, or downregulating the expression of cell surface molecules that phage use as receptors during the first stage of infection (Charbit et al., 1984; Høyland‐Kroghsbo et al., 2013; Laanto et al., 2012; Meyer & Lenski, 2020; Rakhuba et al., 2010). Under certain conditions, phage can overcome this perturbation by evolving to use a new receptor and retain suppression (Borin et al., 2021; Meyer et al., 2012). Therefore, phages with enhanced ability to evolve to use new receptors and expand their host‐range should be more effective at suppressing bacteria, especially over the long term when bacteria can evolve resistance.

Ideally, of course, researchers should select phages that are stable, fast‐reproducing, and evolvable. However, tradeoffs constrain the simultaneous optimization of multiple traits (Beardmore et al., 2023; Edwards et al., 2021; Goldhill & Turner, 2014; Meyer et al., 2015). Indeed, there is a well‐known tradeoff between stability and reproduction that has been demonstrated in RNA viruses (Dessau et al., 2012; Singhal et al., 2017). And tradeoffs between thermodynamic stability and evolvability have been demonstrated for bacteriophage λ (Strobel et al., 2022). If tradeoffs are common in nature, then it may be difficult to identify naturally occurring phages with multiple favorable traits, and it will be helpful to understand which traits to prioritize when selecting phages. Furthermore, with the development of more sophisticated genetic engineering technologies, researchers are increasingly attempting to design phage with desirable characteristics (Favor et al., 2020; Yehl et al., 2019). Such attempts could easily be derailed without a solid understanding of which traits are most important and how tradeoffs constrain trait optimization.

We capitalized on the wealth of knowledge on bacteriophage λ evolution and molecular biology (Casjens & Hendrix, 2015; Meyer et al., 2012) to generate a system to test the importance of evolvability, reproductive rate, and stability. When *Escherichia coli* and λ are cocultured together under certain conditions, they engage in a predictable arms race (Gupta, Peng, et al., 2022; Gupta, Zaman, et al., 2022; Meyer et al., 2012). *E. coli* evolves mutations in a regulatory gene, *malT*, that interferes with expression of λ's receptor, LamB, conferring nearly complete resistance. λ evolves mutations in its host‐recognition protein (J) that allow it to use a new receptor, OmpF. *E. coli* can evolve other resistance mutations, including some in genes *manYZ* that interfere with λ's ability to transport its DNA into the cytoplasm. However, *malt* mutations tend to evolve before *manYZ*. This is because the *malt* gene has a high mutation rate, owing to its repetitive sequence that causes 25‐base duplications. *malt* mutations can additionally confer a slight cost‐saving fitness advantage compared to the costlier *manYZ* mutations (Burmeister et al., 2020; Chaudhry et al., 2018; Meyer et al., 2010; Pelosi et al., 2006).

**FIGURE 1:**
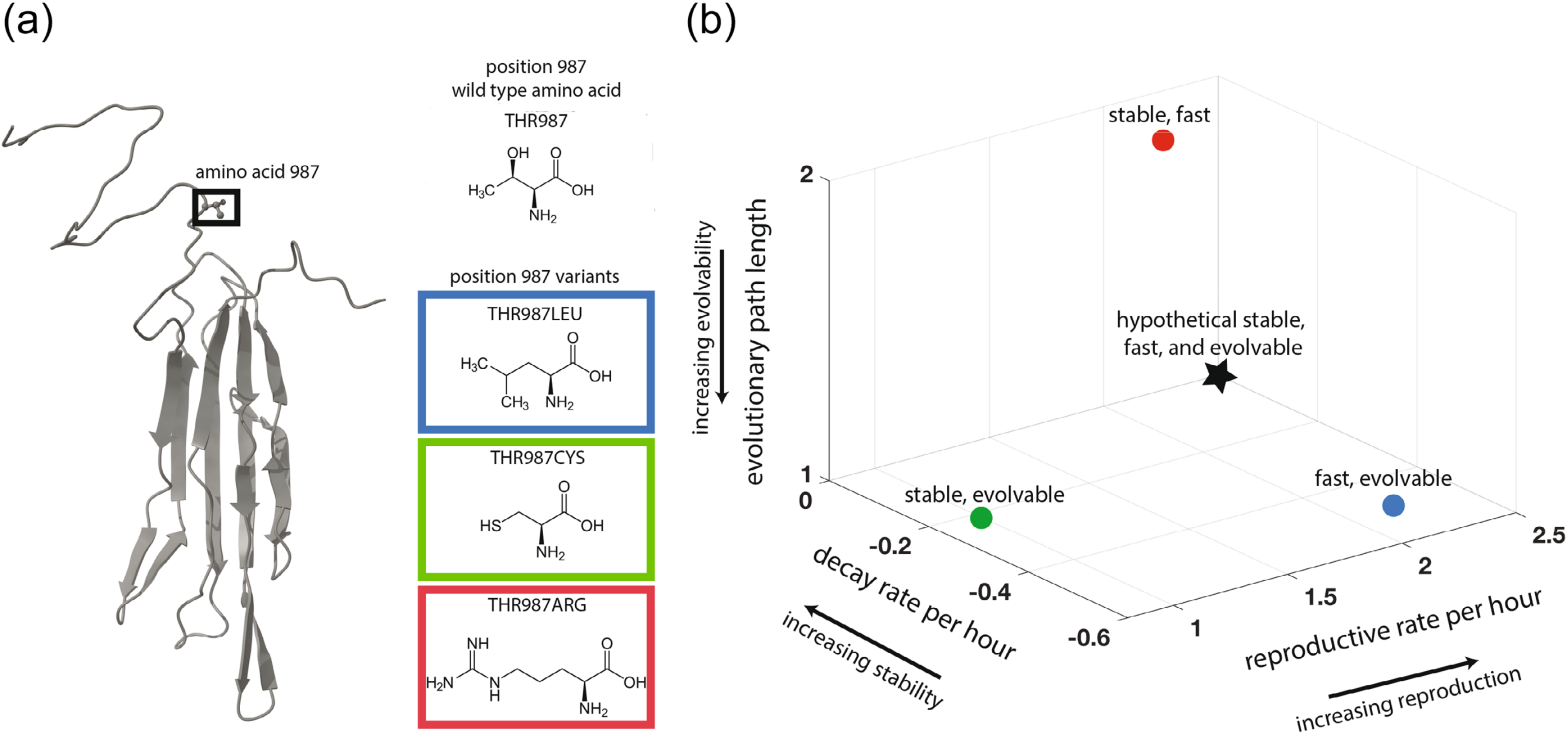
A trio of closely related phage genotypes demonstrate a three‐way tradeoff between stability, reproduction, and evolvability. Panel a: AlphaFold prediction of the domain of the λ receptor binding protein that determines host range. The three λ genotypes in this study were identical except for a single amino acid difference in this domain. Insets show the wild type amino acid and three variant amino acids. Panel b: Three‐dimensional plot of phage trait values. Stability is measured by decay rate, the rate at which phage lose infectivity in an environment lacking hosts. Reproduction is measured by reproductive rate, the rate at which phage replicate on their host bacteria, adjusted to account for the phage lost to decay. Evolutionary path length is the number of mutations required to infect through the non‐native receptor. It is the inverse of evolvability because a genotype requiring fewer mutations to achieve a new function is more evolvable. The position on the graph corresponding to optimality of all three traits is indicated by the star in the lower back corner. No phage was able to optimize all three traits.

We studied three nearly identical λ genotypes from a previously created library of variants differing only by a single amino acid in the receptor binding protein J (Strobel et al., 2022). The initial library contained nine variants, and none exhibited optimal values for reproductive rate, thermodynamic stability, or speed of evolving the counter defense to use a new receptor (OmpF). Perhaps surprisingly, given their high genotypic similarity, they exhibited significant phenotypic differences across our three traits of interest, and we chose three genotypes that had each optimized a different pair of traits (Figure 1). Two strains were evolvable, which in the context of this study meant that they were just one mutation away from evolving to use the new receptor, OmpF, which is the first counter‐defense trait known to evolve in this well‐studied system. Two genotypes had a fast reproductive rate, doubling their populations about every 20 min, whereas the slower phage takes twice as long. The two thermodynamically stable phages' half‐lives are about 3.5 h, whereas the unstable phage's is 1.25 h.

To test which traits enhance λ's ability to suppress its *Escherichia coli* host, we performed a 10‐day experiment in which each phage genotype was incubated with a bacterial strain sensitive to λ. As a metric of bacterial suppression, we monitored bacterial population size daily. The study was designed such that each trait was optimized in two of the three phages, so if a single trait was most important for suppression, the bacterial density would be significantly lower in the two treatments with phages that possess the trait. If two traits are valuable and are complimentary, or if two traits interact synergistically, then a single treatment would be the most suppressive. And if all characteristics are equally important (or unimportant), then all three treatments will suppress equally. We gave each genotype a short‐hand name for the two traits that it had optimized: the fast‐evolvable genotype had optimized reproductive rate and evolvability, the fast‐stable genotype had optimized reproductive rate and stability, and the stable‐evolvable genotype had optimized stability and evolvability. We found that one phage, the fast‐evolvable genotype, suppressed bacteria more than the others, suggesting evolvability and fast reproduction work together to achieve the greatest suppression. Upon finding this, we tested whether the two traits had independent effects on suppression. To do this, we engineered the fast‐evolvable and stable‐evolvable strains with the mutation necessary to exploit usage of the OmpF receptor, which effectively collapsed the three‐way tradeoff to a pairwise compromise between reproductive rate and stability (Strobel et al., 2022). The newly generated “fast‐evolved” genotype was more suppressive than the “stable‐evolved” genotype suggesting fast reproduction had an effect outside of evolvability (Strobel et al., 2022). For one final study, we tested the environmental contingency of our results by testing strain suppressiveness under conditions that should favor stable phages. We found that this manipulation shifted which phage was most suppressive in the short term, but not in the long term.

## 2 MATERIALS AND METHODS

### 2.1 Media

**FIGURE 2:**
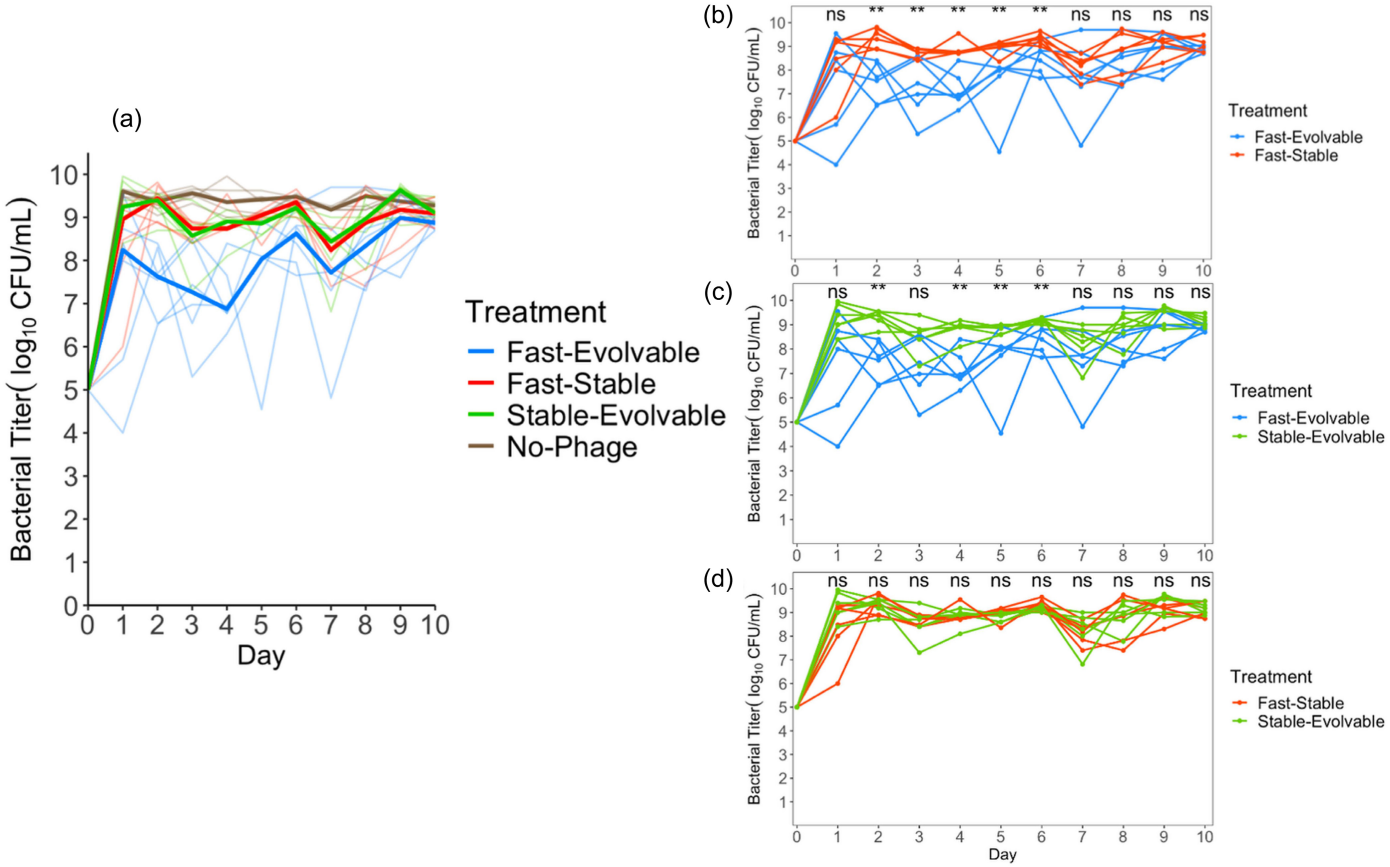
λ suppression of bacteria monitored daily for 10 days. Each line corresponds to the bacterial titer in a single replicate flask population. Six replicate flasks were initiated for each phage genotype. Across all genotypes, the same bacterial strain was used to initiate the flask and approximately the same ratio of phage and bacteria were added. Panel a: All three phage genotypes are shown together. Median lines for bacterial population replicates suppressed by a given genotype are shown in bold, and individual populations are shown by translucent lines. Panel b–d: pairs of genotypes are shown for ease of visualizing differences. Statistical differences are present on days 2–6 between the fast‐evolvable replicates and fast‐stable replicates, days 2, 4, 5, and 6 between the fast‐evolvable replicates and stable‐evolvable replicates, and statistical differences were not detected between the fast‐stable replicates and stable‐evolvable treatments at any time (Table S2). A Wilcoxon rank‐sum test was used to test statistical significance between levels of suppression between two phage genotypes. **p < 0.01.

**FIGURE 3:**
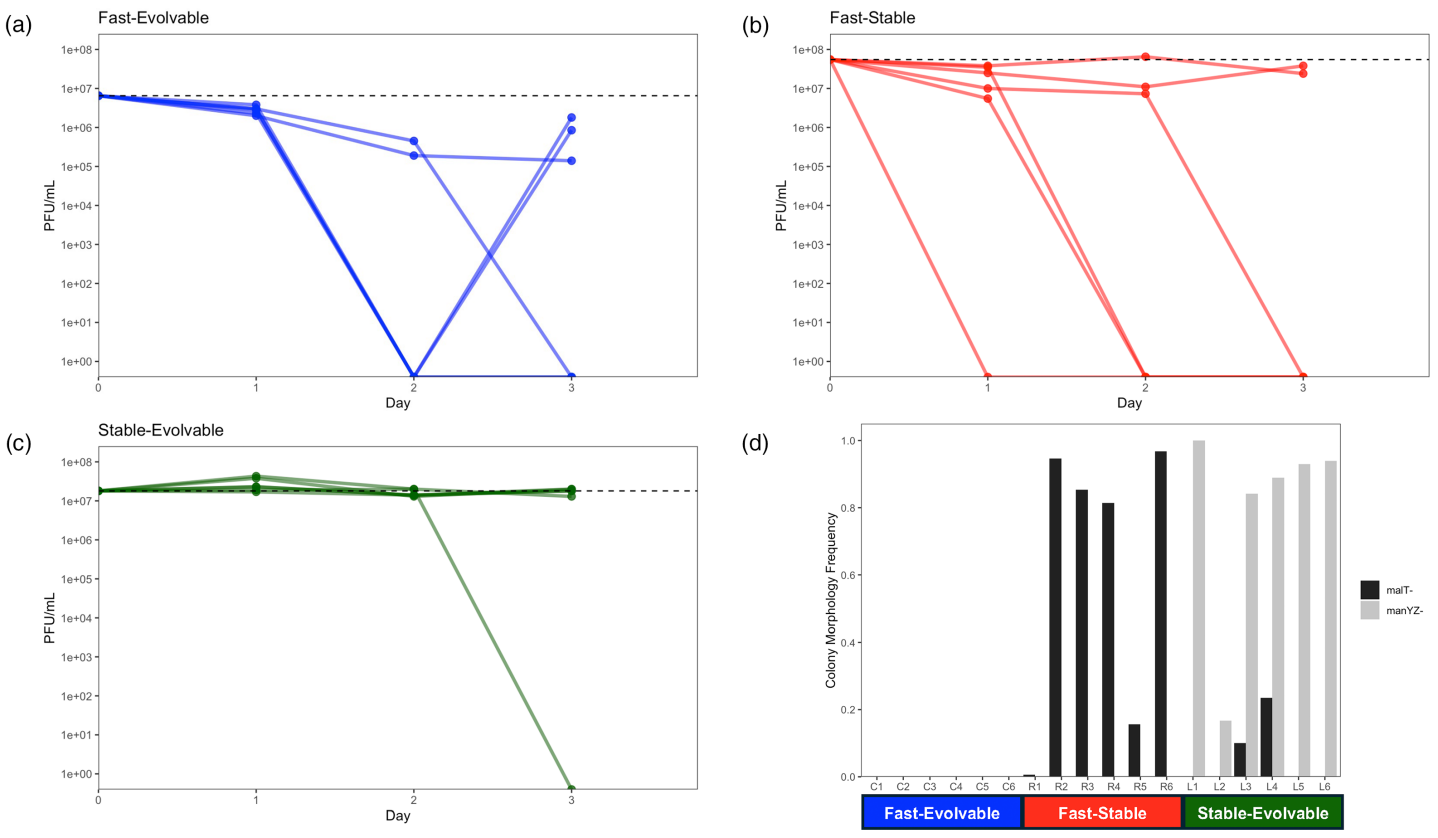
*Escherichia coli* resistance evolution. (a–c) Plaque forming units (PFU) of each phage genotype on bacteria that was cocultured with the phage. The dashed line indicates the PFU measured on the bacterial strain used in the suppression experiments (REL606). A single isolate from each replicate was studied for each day of the experiment through the third day. PFU values lower than the dotted line indicates a gain of resistance, values on the *x*‐axis were cases of complete resistance where no plaques were observed. (d) Frequency of colonies with morphologies in line with possessing resistant mutations in genes *malT* and *manYZ*. All six replicates per treatment were sampled on day two of the experiment.

**FIGURE 4:**
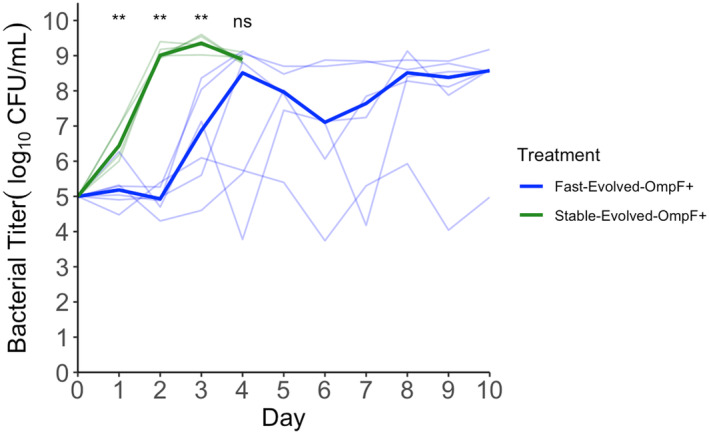
Bacterial population dynamics exposed to two λ genotypes differing only in their reproductive rates. Both genotypes were engineered to contain a key mutation that conferred activity on the OmpF receptor, allowing infection of bacteria that are resistant to OmpF^−^ phage. Six replicate flask populations were initiated for each phage genotype, with the same bacterial strain. Median lines for bacterial population replicates of a given genotype are shown in bold, and individual populations are shown by translucent lines. Each translucent line corresponds to the bacterial titer in a single replicate flask population. The stable, slow reproducing phage (green) poorly suppressed the bacteria and the phages passed below our limit of detection after the first day. The stable‐evolved replicates were discontinued after 3 days of no phage detection. Statistical differences between replicate populations are present on days 1, 2, and 3 (Table S2). A Wilcoxon rank‐sum test was used to test statistical significance between levels of suppression between the two phage genotypes. **p < 0.01.

**FIGURE 5:**
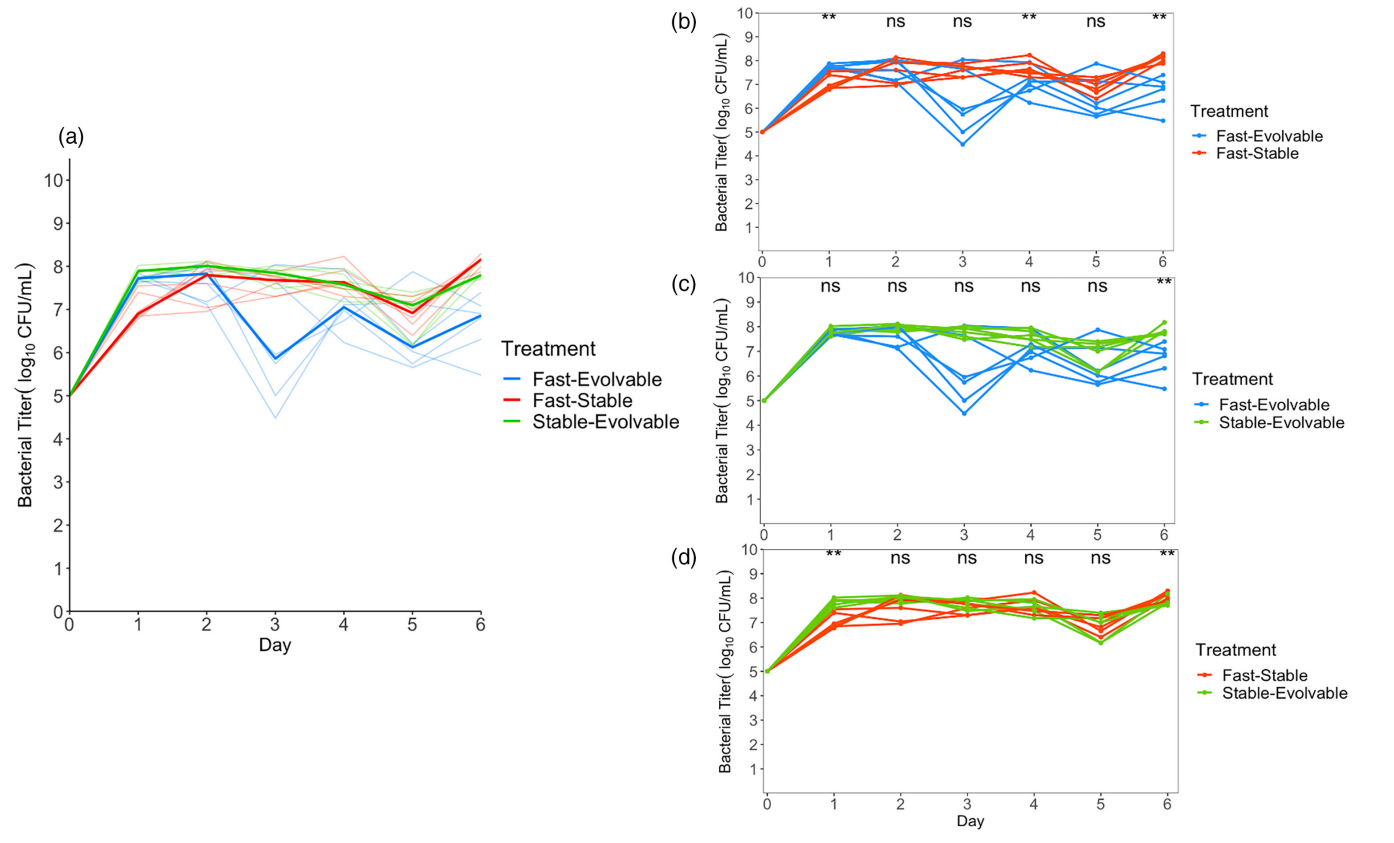
Environmental contingency of bacterial suppression dynamics. The conditions of this experiment were identical to those of Figure 2, except that the available glucose was reduced by 10‐fold, limiting the maximum potential bacterial carrying capacity throughout the duration of the experiment. Panel a: All three phage genotypes are shown together. Median lines for bacterial population replicates suppressed by a given genotype are shown in bold, and individual populations are shown by translucent lines. Panels b–d: pairs of genotypes are shown for ease of visualizing differences. Statistical differences are present on days 1, 4, and 6 between the fast‐evolvable replicates and fast‐stable replicates, day 6 between the fast‐evolvable replicates and stable‐evolvable replicates, and days 1 and 6 between the fast‐stable replicates and stable‐evolvable replicates (Table S2). A Wilcoxon rank‐sum test was used to test statistical significance between levels of suppression between two phage genotypes. **p < 0.01.

Most media were prepared exactly as described in a previous study (Strobel et al., 2022). *E. coli* cultures revived from the freezer were first grown overnight in Lennox Broth (LB). And then recovered for a second day in in M9 Glucose, the medium used for the bacterial suppression experiments (Figures 2, 3, 4, 5). Two different concentrations of glucose were used in the suppression experiments, the typical level, 5.55 mM (Figures 2, 3, 4) and a lower level, 0.55 mM (Figure 5). Recipes for tetrazolium indicator plates can be found in another study (Meyer et al., 2012).

### 2.2 Phage strains

The fast‐evolvable, fast‐stable, and stable‐evolvable λ genotypes used in this study were derived from lysogenic *λ* phage strains of cI857 that were members of a library of phage variants created for a previous study (Strobel et al., 2022). For ease of engineering, the initial library was created by editing a lysogenic λ prophage integrated into the *E. coli* chromosome. However, lysogenic phages have not been approved for therapy because they can cause complications like conferring phage resistance or facilitating horizontal gene transfer within microbiomes (Dedrick et al., 2019). Therefore, we edited each phage genome to be obligatorily lytic by introducing stop codons within the *cI* repressor gene that maintains lysogeny. Gene editing was performed using multiplexed automated genome engineering (MAGE) (Wang et al., 2009; Wang & Church, 2011). We used a slightly modified MAGE protocol than previously described: first, we grew each lysogen overnight at 30°C in LB with carbenicillin 2.64 mM supplemented; then, we inoculated three replicate tubes of 3 mL LB with 100 μL of overnight culture and 4 μL of 100 μg/mL carbenicillin and incubated for 1 h at 30°C. Next, we added 20 μL of 1 M arabinose to each tube and incubated for another hour at 30°C. Aliquots of 1 mL were then pelleted and washed three times with 4°C sterile nanopure water to remove media and salt residue. We resuspended the cell pellet in 50 μL of nanopure water with 5 μM of a 90‐mer DNA oligo added. The oligo was designed to introduce stop codons into the cI gene (CGCACGGTGTTAGATATTTATCCCTTGCGGTGATAGATTTAACGTATGTGAACAAA AAAGTAACCATTAACACAAGAGCAGCTTGAGGAC). After electroporation, we recovered cells in 3 mL LB overnight. Lastly, we plated phages in the supernatant on a bacterial lawn and selected phages that produced clear plaques, which is a visual indicator of lytic mutants.

### 2.3 *Escherichia coli* strains

For the suppression experiments, we used the *E. coli* strain REL606 (Jeong et al., 2009) as the host bacterium. For detection of phages that evolved to use the OmpF receptor during the suppression experiments, we used the *LamB* ^−^ strain (JW3996) from the Keio collection (Baba et al., 2006). The wildtype parent of the Keio collection (BW25113, referred to as “WT” throughout this manuscript) was used for estimating phage titer during the suppression, reproductive rate, and stability assays.

### 2.4 Sanger sequencing

We sequenced the reactive region of the *J* gene (approximately nucleotide positions 2650 to 3399 of 3399 total bases) to identify mutations in genotypes that evolved to be OmpF^+^ in the coevolution experiment. We also sequenced the *cI* gene to verify successful knockouts.

To amplify DNA fragments for sequencing the *J* gene we used primers: Forward 5′ CCT GCG GGC GGT TTT GTC ATT TA; Reverse 5′ CGC ATC GTT CAC CTC TCA CT and with New England Bioscience Q5® Mastermix. Unpurified PCR products were processed and sequenced by Genewiz La Jolla, CA. The reverse primer was used to initiate the sequencing reaction.

For sequencing the *cI* gene we used primers: Forward 5′ CGA CCA GAA CACCTT GCC 3′; Reverse 5′ CCC TTG CGG TGA TAG ATT TAA CG 3′ and sent unpurified PCR products to Genewiz La Jolla, CA, for sequencing with forward and reverse primers. All sequences were aligned to the appropriate reference using Unipro UGENE v1.31.1 (Okonechnikov et al., 2012).

### 2.5 Stability, reproductive rate, and evolvability measurements

Stability and net reproductive rates for the fast‐stable, fast‐evolvable, and stable‐evolvable genotypes were measured in a previous study (Strobel et al., 2022). Stability was measured as the rate at which phage lost infectivity in media alone (i.e. no host cells), and net reproductive rate was measured as the rate of increase in phage titer when incubated with permissive host cells (Strobel et al., 2022). The latter is a combined rate of reproduction and decay, so for the current study we obtained just the rate at which phage reproduce (i.e. factoring out decay) by subtracting the decay rate from the combined rate (i.e. reproductive rate = net reproductive rate − decay rate). Evolvability was measured as the ability of the phage to evolve to use the non‐native OmpF receptor. Several measures of evolvability were previously reported: the number of replicate populations that evolved to use OmpF, the average number of days required to evolve OmpF^+^, or the number of mutations required to use OmpF (Strobel et al., 2022). All metrics were correlated and so here we only report the last metric, evolutionary path length. Fewer mutations, or a shorter evolutionary path, corresponds to higher evolvability (Kirschner & Gerhart, 1998).

For the genotypes that we edited to receive the N1107K mutation (fast‐evolved and stable‐evolved), we measured their net reproductive rates immediately after generating the obligate lytic versions. Following the protocol in (Strobel et al., 2022), we picked a single plaque of each genotype into 100 μL of M9 Glucose and divided the volume of 100 μL into the three replicate 50 mL flasks with M9 Glucose and 0.01 M MgSO_4_. We then measured initial phage titers by diluting the flask contents and plating with WT cells infused in soft agar and added ~10^8^ REL606 cells to each flask and incubated at 37°C shaking for 4 h. The bacteria outnumbered the phages by ~1000‐fold meaning they had ample host cells to reproduce on in the limited 4‐h experiment. We chose this design to eliminate nonlinear host‐density or host‐resistance evolution effects. After incubation, samples were taken from each flask, filtered through 0.22 μM filters to remove bacteria, and phage titers were re‐measured by diluting in M9 glucose + MgSO_4_ and plating with WT cells infused in soft agar. We measured growth over 4 h rather than 24 h (the length of time between transfers in the evolution experiment) to reduce the possibility that genotypes would evolve mutations during the growth experiment. We did not measure decay rates of the fast‐evolved and stable‐evolved genotypes, so we report net reproductive rates for all four genotypes in Figure S4.

Because we measured the net reproductive rates of the fast‐evolvable and fast‐stable in the previous study (lysogenic versions) and here (obligate lytic versions), we were able to assess whether knocking out the *cI* gene to make the obligate lytic versions altered net reproductive rate. We did not find a significant effect (2‐sample *t*‐test, *n* = 3 per genotype; fast‐evolvable: *t*‐stat = −0.110, df = 4, *p* = 0.918; stable‐evolvable: *t*‐stat = 0.566, df = 4, *p* = 0.602).

### 2.6 Bacterial suppression experiments

To determine which phage genotypes would best suppress REL606, we inoculated six 50‐mL flasks per phage genotype with 10 mL modified M9 glucose, 10^6^ bacterial cells, and approximately 10^5^–10^6^ phage particles (exact values reported in Table S2). Flasks were incubated at 37°C, shaking at 120 rpm. After 24 h, 100 μL of each community was transferred into new flasks with 10 mL of fresh media. Flasks were passaged for 10 days for the first two suppression experiments (Figures 2 and 4) and for 6 days in the third suppression experiment (Figure 5) because the phage lost suppressive ability by then in the earlier experiments. Each day, 1 mL aliquots were removed to estimate bacterial and phage densities, as well as to freeze communities with glycerol for later analysis (40 μL 80% glycerol per 200 μL sample). To assess bacterial titers, aliquots were diluted in M9 glucose and spot plated on LB agar. For phages, 1‐mL aliquots were centrifuged (1 min at 15,000 *g*) to pellet cells. Then, supernatants were serially diluted in M9 glucose, and 2‐μL aliquots were spotted on a lawn of REL606 infused in soft agar to obtain phage titers. After the experiment was completed, we ran a no‐phage control identically as the initial suppression experiments, but without the addition of phages and we did not preserve samples daily. We ran the experiment in two blocks of three replicates started on separate days to test whether there were day effects, there were not.

### 2.7 Tracking coevolutionary dynamics during the suppression experiment

We tracked the evolution of resistance with multiple different strategies. First, we isolated a single bacterial isolated from every replicate of every treatment for the first 3 days of the experiment. This time period was chosen because the bacterial densities varied the most early in the suppression assay. We streaked for colonies on LB plates, then re‐streaked on LB to purify the cells of phage, and then re‐streaked on Tetrazolium Maltose indicator plates to determine if the isolates were *malT* ^*−*^, a common resistance mutation (Meyer et al., 2012). Next, we quantified resistance of each host isolate by measuring efficiency of plaquing (EOP) (Gupta, Peng, et al., 2022) with the initial phage the *E. coli* were co‐cultured with. Lastly, to gain insight on the diversity of resistance within populations, we calculated the frequency of *malT* ^*−*^ and *manYZ* ^*−*^ cells using indicator plates. *manYZ* ^*−*^ is another common mutation that causes λ resistance and identified using tetrazolium mannose indicator plates (Meyer et al., 2012). Bacteria were revived from stocks preserved in the freezer from the second day of the suppression experiment. This day was chosen because it is the first where the bacterial densities begin to diverge. Samples were diluted and plated onto each indicator plate. On average, 54 colonies' phenotypes were scored for whether they had the wild type, *malT* ^*−*^, and/or *manYZ* ^*−*^ phenotype for each replicate.

To track counter‐defense evolution, we plated ~5 μL of cryopreserved samples from every treatment and replicate on day 2, onto lawns of *lamB* ^*−*^ *E. coli*. We spotted two additional lysates from a phage reliant on LamB (cI26) and an OmpF^+^ λ (EvoC) (for a description of the λ strains see Meyer et al., 2012). This was done to test which populations had evolved to counteract *malT* ^*−*^ *E. coli*.

### 2.8 Bioinformatic prediction of the J structure

We used the publicly available version (Mirdita et al., 2022) of Alphafold (Jumper et al., 2021) to predict the structure of the reactive region of the wild type J domain containing the 173 most C‐terminal amino acids. We used Chimera to create Figure 1a (Pettersen et al., 2004).

### 2.9 Statistical tests and plotting

We used RStudio (RStudio Team, 2020) to create Figures 2, 3, 4, 5, Figures S1, S3, and S5 and to carry out statistical tests in this manuscript. We used a two‐sided Wilcoxon rank‐sum test and *α* value of 0.05 for statistical significance between replicates of pairs of genotypes on a single day. We used MATLAB to create Figure 1b and Figure S4. For statistical comparisons of trait values in Figure S4, we conducted two‐sample *t*‐tests after verifying equal variances in MATLAB.

## 3 RESULTS

### 3.1 Bacterial suppression

Of the three phage genotypes, the fast‐evolvable proved to be most suppressive (Figure 2a, Figure S1). There are statistical differences in the bacterial densities between the fast‐evolvable and fast‐stable genotype on days 2, 3, 4, 5, and 6 and between the fast‐evolvable and fast‐stable genotype on days 2 4, 5, and 6 (Table S3; Figure 2b,c). The other two genotypes, fast‐stable and stable‐evolvable, appear to be equally poor at suppressing, and the bacterial densities were not statistically different from each other on any day (Table S3; Figure 2d). Although there are timepoints where there are not significant differences in suppression, at least one replicate of fast‐evolvable was the most suppressive on all 10 days of the experiment (Figure 2b,c). Because two of the three genotypes did not suppress much more than the no‐phage control, it is difficult to draw conclusions about all three traits, but stability is not as important for suppression as the other two traits.

### 3.2 Coevolution during the suppression trial

We hypothesized that the difference in suppression between the treatments was in part due to differences in coevolutionary dynamics, the speed *E. coli* evolves *malT* ^*−*^ resistance and how fast λ evolves to use OmpF. To test this, we tracked resistance and OmpF^+^ evolution early in the suppression experiment when there were the most significant treatment differences.

For the stable‐evolvable treatment, we found that only one replicate had evolved resistance by the third day (Figure 3a) and the single resistant isolate had a plating phenotype in line with gaining a *malT* ^−^ mutation. The lack of resistance evolution suggests that this phage's inability to suppress was not due to resistance, but likely its slow reproductive rate. A deeper look into resistance evolution via *malT* ^−^ and *manYZ* ^*−*^ on day two revealed no resistant mutants in any replicates of this treatment (Figure 3d). Despite being evolvable, the phage did not evolve to be OmpF^+^ (Figure S2) because there was no need for the phage to evolve counter defenses.

The *E. coli* cocultured with the fast‐stable λ evolved complete resistance in four out of six populations within the first 3 days (Figure 3b). There was a high correspondence between resistant colonies and the *malT* ^*−*^ phenotype (Pearson's correlation = 0.913 *p*‐value = 5.037e‐10), suggesting that resistance evolved through mutations in *malT*. An extensive survey of resistance on day two revealed high frequencies of *malT* ^*−*^ phenotype and no evidence of resistance through *manYZ* ^*−*^ mutations (Figure 3d). Despite having high levels of *malT* ^*−*^ mutants, this less evolvable λ had not evolved to counteract resistance through gaining use of OmpF (Figure S2). λ was unable to suppress this population because it rapidly evolved high levels of resistance.

The coevolutionary dynamics with the fast‐evolvable strain were significantly different and help explain why this phage was the only one capable of suppression. Resistance evolved in all the treatments, but it often only provided partial resistance (Figure 3c). The resistance was not correlated with the *malT* ^*−*^ phenotype suggesting that other loci were evolving resistance mutations (Pearson Correlation = 0.348 *p*‐value = 0.096). A survey of hundreds of colonies per replicate on the second day revealed low levels of *malT* ^*−*^ colony morphs, and high levels of *manYZ* ^*−*^ (Figure 3d). A population‐level assay of OmpF‐use revealed that λ from all six replicates of fast‐evolvable phage evolved to be OmpF^+^, explaining why *malT* ^*−*^ were less common (Figure S2). Fast‐evolvable λ was likely more suppressive because it was able to counteract *malT* ^*−*^ and cause the bacteria to rely on *manYZ* ^*−*^ and other resistance mutations that likely have a slower mutation rate and some of which only provide partial resistance.

Together, these results suggest that having a fast reproductive rate is critical to suppress bacterial populations so that the phage can achieve population levels that can impose top‐down control. Likewise, evolvability is also important to counteract resistance and maintain the ability to suppress bacteria.

### 3.3 Reproductive rate and evolvability independence and synergism

Next, we explored whether the effects of reproductive rate and evolvability to suppress bacterial populations were interdependent, or if we could detect independent effects. We developed an experiment capable of testing whether having a high reproductive rate alone would increase suppression. Our idea was to edit in a mutation (N1107K) that conferred the ability to use OmpF in both the fast‐evolvable and stable‐evolvable genotypes (Strobel et al., 2022). By doing so, we hoped to remove evolvability from the equation by artificially making both genotypes ‘evolved’ with respect to OmpF use. The initial suppression experiment was then repeated with the two evolved genotypes, and the fast‐evolved genotype was substantially more suppressive than the stable‐evolved genotype (Figure 4; Table S3). In fact, not only did the stable‐evolved genotype not suppress the bacteria, it did not grow fast enough to keep up with the dilution from the daily transfer, and every replicate of this genotype lost phage entirely after day two (Figure S3). Because the stable‐evolved genotype was even worse at suppressing than the stable‐evolvable (i.e., before receiving the OmpF^+^ granting mutation), we measured the growth rates of the evolved versions of each genotype to verify that N1107K did not introduce an unexpected fitness cost in the stable‐evolvable background. It did not, although it did cause a significant gain in reproductive rate (combined reproductive rate + decay rate) in the fast‐evolvable background that was not seen in the stable‐evolvable background (Figure S4). Together, these results indicate that reproductive rate has an independent effect on suppression, and evolvability on its own does not confer high suppression because this trait is superfluous if the phage is unable to exert pressure on the host to evolve resistance.

### 3.4 Density‐dependent suppression effects

Thus far, these results suggest that evolvability and reproductive rate are more important for suppression than stability. One possible explanation for this result is that the laboratory environment of our experiments is artificially permissive of instability, compared to the more challenging environment where therapeutic phage would need to be deployed, such as inside a patient's body. In our experiment, phage had access to a homogeneous population of rapidly growing hosts. Inside a patient's body, by contrast, phage would need to survive in a spatially complex environment in which the phage would spend more time outside their host searching for their next host. In that environment, stability might be a better predictor of suppression. To evaluate this hypothesis, we repeated the suppression experiment exactly as was done in Figure 2, except we decreased the amount of glucose, a limiting resource of the bacteria, by 10‐fold. This lowered the carrying capacity of the bacteria in the flask, thereby decreasing the density of host cells, and increasing the amount of time that phages spent in the external environment between hosts, undergoing decay. We hypothesized that under these more challenging conditions, the fast‐evolvable phage might not be more suppressive than the fast‐stable phage. Consistent with expectations, the fast‐stable phage is the most suppressive after 1 day (Figure 5; Figure S5; Table S3). However, by day 3, two‐thirds of the replicates of the fast‐evolvable phage were the most suppressive, and by the end of the experiment, it was all six replicates (Figure 5a–c; Table S3). This finding suggests that evolvability is more important than stability for suppressing bacteria over the long term, even in the more challenging condition where stability should be favored.

## 4 DISCUSSION

The aim of this study was to evaluate which phage trait is most predictive of bacterial suppression. We hypothesized that evolvability would be most predictive, and therefore the fast‐evolvable and stable‐evolvable genotypes would be most suppressive. This assumed that phage fail to suppress because bacteria evolve resistance. The first suppression experiment with all three genotypes revealed instead that a single phage, the fast‐evolvable genotype, was suppressive, while the fast‐stable and stable‐evolvable genotypes were not. This somewhat unexpected result validated the importance of evolvability over stability but also suggested that a fast reproductive rate, in addition to evolvability, is critical for suppression. Our assumption was wrong, phage also fail to suppress if their reproductive rate is too slow. A second experiment confirmed the importance of reproductive rate by comparing strains that possessed a single tradeoff between reproductive rate and stability and finding the fast‐growing but unstable strain was most suppressive. Finally, having shown that stability was the least predictive of suppression, we asked whether our experimental design favored that outcome by using conditions that are artificially permissive to unstable genotypes. To alter our experiment to better reflect the more challenging environment with fewer permissive hosts, we repeated the first suppression experiment using only 10% of the glucose, limiting the bacterial carrying capacity of the flask. Initially, the fast‐stable phage was most suppressive, validating the hypothesis that decreasing host availability penalized unstable phages. However, by the end of the experiment the fast‐stable phage had become non‐suppressive, while the fast‐evolvable phage had become suppressive. We confirmed that the gain in suppression was due to the fast‐growing evolvable phage achieving robust growth on OmpF much earlier than the other genotypes, thereby giving that genotype access to hosts even after they had evolved resistance to the initial phage genotypes. These findings emphasize the importance of considering not only the current traits of therapeutic phages, but also their capacity for adaptive evolution. The reversal of suppression ability across a multi‐day timescale suggests the importance of measuring suppression over an entire course of treatment since resistance and counter‐resistance traits only take days to evolve.

Although the need to consider the future evolutionary potential (i.e. evolvability) has not yet permeated in vivo phage therapy practice, numerous studies have demonstrated that providing an evolutionary advantage to phage generates more suppression. In a technique called ‘phage training’, the evolutionary advantage comes from coevolving a candidate phage with the target bacterium, allowing an evolutionary arms race to play out, and then isolating an evolved phage strain for use against the naïve bacteria (Borin et al., 2021, 2023; Eskenazi et al., 2022; Laanto et al., 2020). Another strategy aims to enhance the evolution of more suppressive phage by increasing opportunities for recombination among different strains, allowing beneficial mutations to be shuffled into a single, highly suppressive genotype (Burrowes et al., 2019). Our study shows that substantial variation in evolvability is possible, even among closely related genotypes. Relatively simple laboratory experiments on existing collections of phages might identify genotypes with unusually high evolutionary potential.

With the rise of genetic engineering technology, there is increasing interest in designing phage with the traits deemed desirable for suppression. Our study sheds light on some possible pitfalls that might frustrate such attempts. One potential pitfall would be failing to consider pleiotropy. In the phage variant library codon 987 was targeted because it had mutated during selection for increased stability (Strobel et al., 2022). Seeking additional variants with a range of stabilities, different amino acids were edited in at codon 987. The assumption was that altering the amino acid at codon 987 affected only stability and evolvability without pleiotropic effects on other traits. This appeared true for most variants; however, the 987CYS (the stable‐evolvable genotype used in this study) had a markedly reduced reproductive rate. This result demonstrates that a genotype that seems to “break” one tradeoff (stability vs. evolvability) might pay a cost in another trait (reproductive rate). Had multiple axes of variation not been considered, this seemingly tradeoff‐breaking phage might have appeared to be an ideal therapeutic phage, but the current study demonstrated the opposite result. Better outcomes might be achieved from ‘bioprospecting’ naturally occurring phages or using directed evolution to evolve enhanced genotypes, rather than attempting to design optimal genotypes, because natural selection should penalize genotypes with low fitness.

There are several limitations to the current study. First, we examined only three phage genotypes, and their genomes were identical except for a single codon. The evolutionary history and idiosyncrasies of this experimental system could prevent our results from being applied generally across the vast diversity of phages. In λ, there is a known tradeoff between stability and evolvability that might not exist in other phages or might be less pronounced (Strobel et al., 2022). In other phages, different combinations of traits might produce tradeoffs, and our study provides a general framework for understanding how tradeoffs limit the optimization of therapeutic phages. Another limitation is that we studied suppression dynamics in flasks under controlled laboratory conditions and used a single phage and a single bacterial host. These conditions are unlike the environment in which therapeutic phages would be deployed. The human gut, for example, where λ‐like phages might be used, is replete with myriad host‐associated microbes in addition to the target, many of which would likely be unavailable as prey for the therapeutic phage (Lozupone et al., 2012). We began exploring this dimension with our limited glucose environment, and our results were robust to the perturbation, but we did not test the effect of non‐host microbes. Phage may also encounter extremes of temperature, pH, and chemicals in vivo that would further penalize unstable genotypes (Blazanin et al., 2022). Despite these differences between λ's natural environment and laboratory conditions, comparisons between laboratory and natural populations of λ revealed that the sites that receive mutations that drive host‐range expansion in the laboratory are present in natural populations, suggesting that laboratory studies are informative for understanding natural dynamics (Maddamsetti et al., 2018). Prior work specific to phage therapy suggests that despite differences between in vitro and in vivo conditions, similar evolutionary dynamics can play out in vitro and in vivo, validating the use of in vitro experiments to inform clinical practices (Castledine et al., 2022).

This work demonstrates the important role of understanding evolutionary biology in phage therapy. Although phage have some properties that are like chemical therapeutics, it is critical to remember that they are biological entities and have their own ability to propagate and evolve. When using directed evolution to create phage therapeutics, phage must be selected to evolve the properties that enable them to suppress bacteria while considering that evolving or engineering one desirable phenotypic characteristic may come at the cost of sacrificing another characteristic. Tradeoffs are difficult to avoid in biological systems, so evaluating traits in the context of tradeoffs is necessary. Considering evolutionary potential alongside conventional attributes, like stability and reproductive rate, allowed us to predict the genotype that best suppressed bacterial population across a multi‐day timeframe.

## CONFLICT OF INTEREST STATEMENT

The authors declare that there are no conflicts of interest.

## Supporting information


Appendix S1

## Data Availability

The data are available through DRYAD at https://doi.org/10.5061/dryad.tmpg4f56w.

## References

[eva13742-bib-0001] Baba, T. , Ara, T. , Hasegawa, M. , Takai, Y. , Okumura, Y. , Baba, M. , Datsenko, K. A. , Tomita, M. , Wanner, B. L. , & Mori, H. (2006). Construction of *Escherichia coli* K‐12 in‐frame, single‐gene knockout mutants: The Keio collection. Molecular Systems Biology, 2, 2006.0008. 10.1038/msb4100050 PMC1681482 16738554

[eva13742-bib-0002] Batinovic, S. , Wassef, F. , Knowler, S. A. , Rice, D. T. F. , Stanton, C. R. , Rose, J. , Tucci, J. , Nittami, T. , Vinh, A. , Drummond, G. R. , Sobey, C. G. , Chan, H. T. , Seviour, R. J. , Petrovski, S. , & Franks, A. E. (2019). Bacteriophages in natural and artificial environments. Pathogens, 8, 100. 10.3390/pathogens8030100 31336985 PMC6789717

[eva13742-bib-0003] Beardmore, R. , Hewlett, M. , Peña‐Miller, R. , Gudelj, I. , & Meyer, J. R. (2023). Canonical host‐pathogen trade‐offs subverted by mutations with dual benefits. The American Naturalist, 201, 659–679. 10.1086/723413 37130231

[eva13742-bib-0004] Blair, J. , Webber, M. , Baylay, A. , Ogbolu, D. , & Piddock, L. (2015). Molecular mechanisms of antibiotic resistance. Nature Reviews Microbiology, 13, 42–51. 10.1038/nrmicro3380 25435309

[eva13742-bib-0005] Blazanin, M. , Lam, W. , Vasen, E. , Chan, B. , & Turner, P. (2022). Decay and damage of therapeutic phage OMKO1 by environmental stressors. PLoS One, 17, e0263887. 10.1371/journal.pone.0263887 35196336 PMC8865689

[eva13742-bib-0006] Bono, L. , Mao, S. , Done, R. E. , Okamoto, K. W. , Chan, B. K. , & Turner, P. E. (2021). Advancing phage therapy through the lens of virus host‐breadth and emergence potential. Advances in Virus Research, 111, 63–110. 10.1016/bs.aivir.2021.07.004 34663499

[eva13742-bib-0007] Borin, J. M. , Avrani, S. , Barrick, J. E. , Petrie, K. L. , & Meyer, J. R. (2021). Coevolutionary phage training leads to greater bacterial suppression and delays the evolution of phage resistance. Proceedings of the National Academy of Sciences of the United States of America, 118, e2104592118. 10.1073/pnas.2104592118 34083444 PMC8201913

[eva13742-bib-0008] Borin, J. M. , Lee, J. J. , Gerbino, K. R. , & Meyer, J. R. (2023). Comparison of bacterial suppression by phage cocktails, dual‐receptor generalists, and coevolutionarily trained phages. Evolutionary Applications, 16, 152–162. 10.1111/eva.13518 36699129 PMC9850009

[eva13742-bib-0009] Broncano‐Lavado, A. , Santamaría‐Corral, G. , Esteban, J. , & García‐Quintanilla, M. (2021). Advances in bacteriophage therapy against relevant multidrug‐resistant pathogens. Antibiotics, 10, 672. 10.3390/antibiotics10060672 34199889 PMC8226639

[eva13742-bib-0010] Brown, E. , & Wright, G. (2016). Antibacterial drug discovery in the resistance era. Nature, 529, 336–343. 10.1038/nature17042 26791724

[eva13742-bib-0011] Bull, J. , Levin, B. , & Molineux, I. (2019). Promises and pitfalls of in vivo evolution to improve phage therapy. Viruses, 11, 1083. 10.3390/v11121083 31766537 PMC6950294

[eva13742-bib-0012] Burmeister, A. R. , Sullivan, R. M. , & Lenski, R. E. (2020). Fitness costs and benefits of resistance to phage lambda in experimentally evolved *Escherichia coli* . In W. Banzhaf , B. H. C. Cheng , K. Deb , K. E. Holekamp , R. E. Lenski , C. Ofria , R. T. Pennock , W. F. Punch , & D. J. Whittaker (Eds.), Evolution in action: Past, present and future: A festschrift in honor of Erik D. Goodman (pp. 123–143). Springer International Publishing.

[eva13742-bib-0013] Burrowes, B. H. , Molineux, I. J. , & Fralick, J. A. (2019). Directed in vitro evolution of therapeutic bacteriophages: The Appelmans protocol. Viruses, 11, 241. 10.3390/v11030241 30862096 PMC6466182

[eva13742-bib-0014] Casey, E. , van Sinderen, D. , & Mahony, J. (2018). In vitro characteristics of phages to guide ‘Real Life’ phage therapy suitability. Viruses, 10, 163. 10.3390/v10040163 29601536 PMC5923457

[eva13742-bib-0015] Casjens, S. R. , & Hendrix, R. W. (2015). Bacteriophage lambda: Early pioneer and still relevant. Virology, 479–480, 310–330. 10.1016/j.virol.2015.02.010 PMC4424060 25742714

[eva13742-bib-0016] Castledine, M. , Padfield, D. , Sierocinski, P. , Soria Pascual, J. , Hughes, A. , Mäkinen, L. , Friman, V. P. , Pirnay, J. P. , Merabishvili, M. , de Vos, D. , & Buckling, A. (2022). Parallel evolution of *Pseudomonas aeruginosa* phage resistance and virulence loss in response to phage treatment in vivo and in vitro. eLife, 11, e73679. 10.7554/eLife.73679 35188102 PMC8912922

[eva13742-bib-0017] Chan, B. K. , Sistrom, M. , Wertz, J. E. , Kortright, K. E. , Narayan, D. , & Turner, P. E. (2016). Phage selection restores antibiotic sensitivity in MDR *Pseudomonas aeruginosa* . Scientific Reports, 6, 26717. 27225966 10.1038/srep26717 PMC4880932

[eva13742-bib-0018] Charbit, A. , Clement, J. , & Hofnung, M. (1984). Further sequence analysis of the phage lambda receptor site. Possible implications for the organization of the lamB protein in *Escherichia coli* K12. Journal of Molecular Biology, 175, 395–401. 10.1016/0022-2836(84)90355-3 6374160

[eva13742-bib-0019] Chaudhry, W. N. , Pleška, M. , Shah, N. N. , Weiss, H. , McCall, I. C. , Meyer, J. R. , Gupta, A. , Guet, C. C. , & Levin, B. R. (2018). Leaky resistance and the conditions for the existence of lytic bacteriophage. PLoS Biology, 16(8), e2005971. 10.1371/journal.pbio.2005971 30114198 PMC6112682

[eva13742-bib-0020] Dedrick, R. M. , Guerrero‐Bustamante, C. A. , Garlena, R. A. , Russell, D. A. , Ford, K. , Harris, K. , Gilmour, K. C. , Soothill, J. , Jacobs‐Sera, D. , Schooley, R. T. , Hatfull, G. F. , & Spencer, H. (2019). Engineered bacteriophages for treatment of a patient with a disseminated drug‐resistant *Mycobacterium abscessus* . Nature Medicine, 25, 730–733. 10.1038/s41591-019-0437-z PMC6557439 31068712

[eva13742-bib-0021] Dessau, M. , Goldhill, D. , McBride, R. , Turner, P. , & Modis, Y. (2012). Selective pressure causes an RNA virus to trade reproductive fitness for increased structural and thermal stability of a viral enzyme. PLoS Genetics, 8, e1003102. 10.1371/journal.pgen.1003102 23209446 PMC3510033

[eva13742-bib-0022] Donadio, S. , Maffioli, S. , Monciardini, P. , Sosio, M. , & Jabes, D. (2010). Antibiotic discovery in the twenty‐first century: Current trends and future perspectives. The Journal of Antibiotics, 63, 423–430. 10.1038/ja.2010.62 20551985

[eva13742-bib-0023] Edwards, K. , Steward, G. , & Schvarcz, C. (2021). Making sense of virus size and the tradeoffs shaping viral fitness. Ecology Letters, 24, 363–373. 10.1111/ele.13630 33146939

[eva13742-bib-0024] Eskenazi, A. , Lood, C. , Wubbolts, J. , Hites, M. , Balarjishvili, N. , Leshkasheli, L. , Askilashvili, L. , Kvachadze, L. , van Noort, V. , Wagemans, J. , Jayankura, M. , Chanishvili, N. , de Boer, M. , Nibbering, P. , Kutateladze, M. , Lavigne, R. , Merabishvili, M. , & Pirnay, J. P. (2022). Combination of pre‐adapted bacteriophage therapy and antibiotics for treatment of fracture‐related infection due to pandrug‐resistant *Klebsiella pneumoniae* . Nature Communications, 13, 302. 10.1038/s41467-021-27656-z PMC8766457 35042848

[eva13742-bib-0025] Favor, A. , Llanos, C. , Youngblut, M. , & Bardales, J. (2020). Optimizing bacteriophage engineering through an accelerated evolution platform. Scientific Reports, 10, 13981. 10.1038/s41598-020-70841-1 32814789 PMC7438504

[eva13742-bib-0026] Goldhill, D. , & Turner, P. (2014). The evolution of life history trade‐offs in viruses. Current Opinion in Virology, 8, 79–84. 10.1016/j.coviro.2014.07.005 25087040

[eva13742-bib-0027] Gordillo, A. F. L. , & Barr, J. (2019). Phage therapy in the postantibiotic era. Clinical Microbiology Reviews, 32, e00066‐18. 10.1128/CMR.00066-18 30651225 PMC6431132

[eva13742-bib-0028] Gupta, A. , Peng, S. , Leung, C. Y. , Borin, J. M. , Medina, S. J. , Weitz, J. S. , & Meyer, J. R. (2022a). Leapfrog dynamics in phage‐bacteria coevolution revealed by joint analysis of cross‐infection phenotypes and whole genome sequencing. Ecology Letters, 25(4), 876–888. 10.1111/ele.13965 35092147 PMC10167754

[eva13742-bib-0029] Gupta, A. , Zaman, L. , Strobel, H. M. , Gallie, J. , Burmeister, A. R. , Kerr, B. , Tamar, E. S. , Kishony, R. , & Meyer, J. R. (2022b). Host‐parasite coevolution promotes innovation through deformations in fitness landscapes. eLife, 11, e76162. 10.7554/eLife.76162 35793223 PMC9259030

[eva13742-bib-0030] Hejnowicz, M. , Gągała, U. , Weber‐Dąbrowska, B. , Węgrzyn, G. , & Dadlez, M. (2014). Phage therapy: Current research and applications. Caister Academic Press.

[eva13742-bib-0031] Høyland‐Kroghsbo, N. , Maerkedahl, R. , & Svenningsen, S. (2013). A quorum‐sensing‐induced bacteriophage defense mechanism. mBio, 4, e00312. 10.1128/mBio.00362-12 PMC3624510 23422409

[eva13742-bib-0032] Hyman, P. (2019). Phages for phage therapy: Isolation, characterization, and host range breadth. Pharmaceuticals, 12, 35. 10.3390/ph12010035 30862020 PMC6469166

[eva13742-bib-0033] Jeong, H. , Barbe, V. , Lee, C. H. , Vallenet, D. , Yu, D. S. , Choi, S. H. , Couloux, A. , Lee, S. W. , Yoon, S. H. , Cattolico, L. , Hur, C. G. , Park, H. S. , Ségurens, B. , Kim, S. C. , Oh, T. K. , Lenski, R. E. , Studier, F. W. , Daegelen, P. , & Kim, J. F. (2009). Genome sequences of *Escherichia coli* B strains REL606 and BL21(DE3). Journal of Molecular Biology, 394, 644–652. 10.1016/j.jmb.2009.09.052 19786035

[eva13742-bib-0034] Jumper, J. , Evans, R. , Pritzel, A. , Green, T. , Figurnov, M. , Ronneberger, O. , Tunyasuvunakool, K. , Bates, R. , Žídek, A. , Potapenko, A. , Bridgland, A. , Meyer, C. , Kohl, S. A. A. , Ballard, A. J. , Cowie, A. , Romera‐Paredes, B. , Nikolov, S. , Jain, R. , Adler, J. , … Hassabis, D. (2021). Highly accurate protein structure prediction with AlphaFold. Nature, 596, 583–589. 10.1038/s41586-021-03819-2 34265844 PMC8371605

[eva13742-bib-0035] Jurczak‐Kurek, A. , Gąsior, T. , Nejman‐Faleńczyk, B. , Bloch, S. , Dydecka, A. , Topka, G. , Necel, A. , Jakubowska‐Deredas, M. , Narajczyk, M. , Richert, M. , Mieszkowska, A. , Wróbel, B. , Węgrzyn, G. , & Węgrzyn, A. (2016). Biodiversity of bacteriophages: Morphological and biological properties of a large group of phages isolated from urban sewage. Scientific Reports, 6, 34338. 10.1038/srep34338 27698408 PMC5048108

[eva13742-bib-0036] Kirschner, M. , & Gerhart, J. (1998). Evolvability. Proceedings of the National Academy of Sciences of the United States of America, 95, 8420–8427. 10.1073/pnas.95.15.8420 9671692 PMC33871

[eva13742-bib-0037] Kutter, E. , de Vos, D. , Gvasalia, G. , Alavidze, Z. , Gogokhia, L. , Kuhl, S. , & Abedon, S. (2010). Phage therapy in clinical practice: Treatment of human infections. Current Pharmaceutical Biotechnology, 11, 69–86. 10.2174/138920110790725401 20214609

[eva13742-bib-0038] Laanto, E. , Bamford, J. , Laakso, J. , & Sundberg, L. R. (2012). Phage‐driven loss of virulence in a fish pathogenic bacterium. PLoS One, 7, e53157. 10.1371/journal.pone.0053157 23308090 PMC3534065

[eva13742-bib-0039] Laanto, E. , Mäkelä, K. , Hoikkala, V. , Ravantti, J. , & Sundberg, L. (2020). Adapting a phage to combat phage resistance. Antibiotics, 9, 291. 10.3390/antibiotics9060291 32486059 PMC7345892

[eva13742-bib-0040] Labrie, S. , Samson, J. , & Moineau, S. (2010). Bacteriophage resistance mechanisms. Nature Reviews Microbiology, 8, 317–327. 10.1038/nrmicro2315 20348932

[eva13742-bib-0041] Lozupone, C. , Stombaugh, J. , Gordon, J. , Jansson, J. , & Knight, R. (2012). Diversity, stability and resilience of the human gut microbiota. Nature, 489, 220–230. 10.1038/nature11550 22972295 PMC3577372

[eva13742-bib-0042] Maddamsetti, R. , Johnson, D. T. , Spielman, S. J. , Petrie, K. L. , Marks, D. S. , & Meyer, J. R. (2018). Gain‐of‐function experiments with bacteriophage lambda uncover residues under diversifying selection in nature. Evolution, 72, 2234–2243. 10.1111/evo.13586 30152871 PMC6646904

[eva13742-bib-0043] Meyer, J. R. , Agrawal, A. A. , Quick, R. T. , Dobias, D. T. , Schneider, D. , & Lenski, R. E. (2010). Parallel changes in host resistance to infection during 45,000 generations of relaxed selection. Evolution, 64(10), 3024–3034. 10.1111/j.1558-5646.2010.01049.x 20550574

[eva13742-bib-0044] Meyer, J. R. , Dobias, D. T. , Weitz, J. S. , Barrick, J. E. , Quick, R. T. , & Lenski, R. E. (2012). Repeatability and contingency in the evolution of a key innovation in phage lambda. Science, 335, 428–432. 10.1126/science.1214449 22282803 PMC3306806

[eva13742-bib-0045] Meyer, J. R. , Gudelj, I. , & Beardmore, R. (2015). Biophysical mechanisms that maintain biodiversity through trade‐offs. Nature Communications, 6, 6278. 10.1038/ncomms7278 25695944

[eva13742-bib-0046] Meyer, J. R. , & Lenski, R. E. (2020). In W. Banzhaf , B. H. C. Cheng , C. Ofria , D. J. Whittaker , K. Deb , K. E. Holekamp , R. E. Lenski , R. T. Pennock , & W. F. Punch (Eds.), Evolution in action: Past, present, and future. Springer.

[eva13742-bib-0047] Mirdita, M. , Schütze, K. , Moriwaki, Y. , Heo, L. , Ovchinnikov, S. , & Steinegger, M. (2022). ColabFold – Making protein folding accessible to all. Nature Methods, 19, 679–682. 10.1101/2021.08.15.456425 35637307 PMC9184281

[eva13742-bib-0048] Okonechnikov, K. , Golosova, O. , & Fursov, M. (2012). Unipro UGENE: A unified bioinformatics toolkit. Bioinformatics, 28, 1166–1167. 10.1093/bioinformatics/bts091 22368248

[eva13742-bib-0049] Pelosi, L. , Kühn, L. , Guetta, D. , Garin, J. , Geiselmann, J. , Lenski, R. E. , & Schneider, D. (2006). Parallel changes in global protein profiles during long‐term experimental evolution in *Escherichia coli* . Genetics, 173(4), 1851–1869. 10.1534/genetics.105.049619 16702438 PMC1569701

[eva13742-bib-0050] Pettersen, E. , Goddard, T. D. , Huang, C. C. , Couch, G. S. , Greenblatt, D. M. , Meng, E. C. , & Ferrin, T. E. (2004). UCSF Chimera – A visualization system for exploratory research and analysis. Journal of Computational Chemistry, 25, 1605–1612. 10.1002/jcc.20084 15264254

[eva13742-bib-0051] Rakhuba, D. , Kolomiets, E. , Dey, E. , & Novik, G. (2010). Bacteriophage receptors, mechanisms of phage adsorption and penetration into host cell. Polish Journal of Microbiology, 59, 145–155. 21033576

[eva13742-bib-0052] RStudio Team . (2020). RStudio: Integrated development for R. RStudio. http://www.rstudio.com/

[eva13742-bib-0053] Russell, D. A. , & Hatfull, G. F. (2017). PhagesDB: The actinobacteriophage database. Bioinformatics, 33, 784–786. 10.1093/bioinformatics/btw711 28365761 PMC5860397

[eva13742-bib-0054] Singhal, S. , Leon Guerrero, C. M. , Whang, S. G. , McClure, E. M. , Busch, H. G. , & Kerr, B. (2017). Adaptations of an RNA virus to increasing thermal stress. PLoS One, 12, e0189602. 10.1371/journal.pone.0189602 29267297 PMC5739421

[eva13742-bib-0055] Strobel, H. , Horwitz, E. , & Meyer, J. (2022). Viral protein instability enhances host‐range evolvability. PLoS Genetics, 18, e1010030. 10.1371/journal.pgen.1010030 35176040 PMC8890733

[eva13742-bib-0056] UC San Diego School of Medicine Center for Innovative Phage Applications and Therapeutics . (2024). https://medschool.ucsd.edu/som/medicine/divisions/idgph/research/center‐innovative‐phage‐applications‐and‐therapeutics/Pages/default.aspx

[eva13742-bib-0057] Wang, H. , & Church, G. (2011). Multiplexed genome engineering and genotyping methods applications for synthetic biology and metabolic engineering. Methods in Enzymology, 498, 409–426. 10.1016/B978-0-12-385120-8.00018-8 21601688

[eva13742-bib-0058] Wang, H. , Isaacs, F. J. , Carr, P. A. , Sun, Z. Z. , Xu, G. , Forest, C. R. , & Church, G. M. (2009). Programming cells by multiplex genome engineering and accelerated evolution. Nature, 460, 894–898. 10.1038/nature08187 19633652 PMC4590770

[eva13742-bib-0059] Yehl, K. , Lemire, S. , Yang, A. C. , Ando, H. , Mimee, M. , Torres, M. D. T. , de la Fuente‐Nunez, C. , & Lu, T. K. (2019). Engineering phage host‐range and suppressing bacterial resistance through phage tail fiber mutagenesis. Cell, 179, 459–469.e9. 10.1016/j.cell.2019.09.015 31585083 PMC6924272

[eva13742-bib-0060] Zablocki, O. , Adriaenssens, E. , & Cowan, D. (2015). Diversity and ecology of viruses in hyper arid desert soils. Applied and Environmental Microbiology, 82, 770–777. 10.1128/AEM.02651-15 26590289 PMC4725269

