# Supplementary material for: More evolvable bacteriophages better suppress their host
Source: Evol Appl. 2024 Jul 4;17(7):e13742. doi: 10.1111/eva.13742 (PMC11224127; doi:10.1111/eva.13742)
Supplement: Supplementary file 1 — Appendix S1 [file EVA-17-e13742-s001.docx]

## Supplementary Material


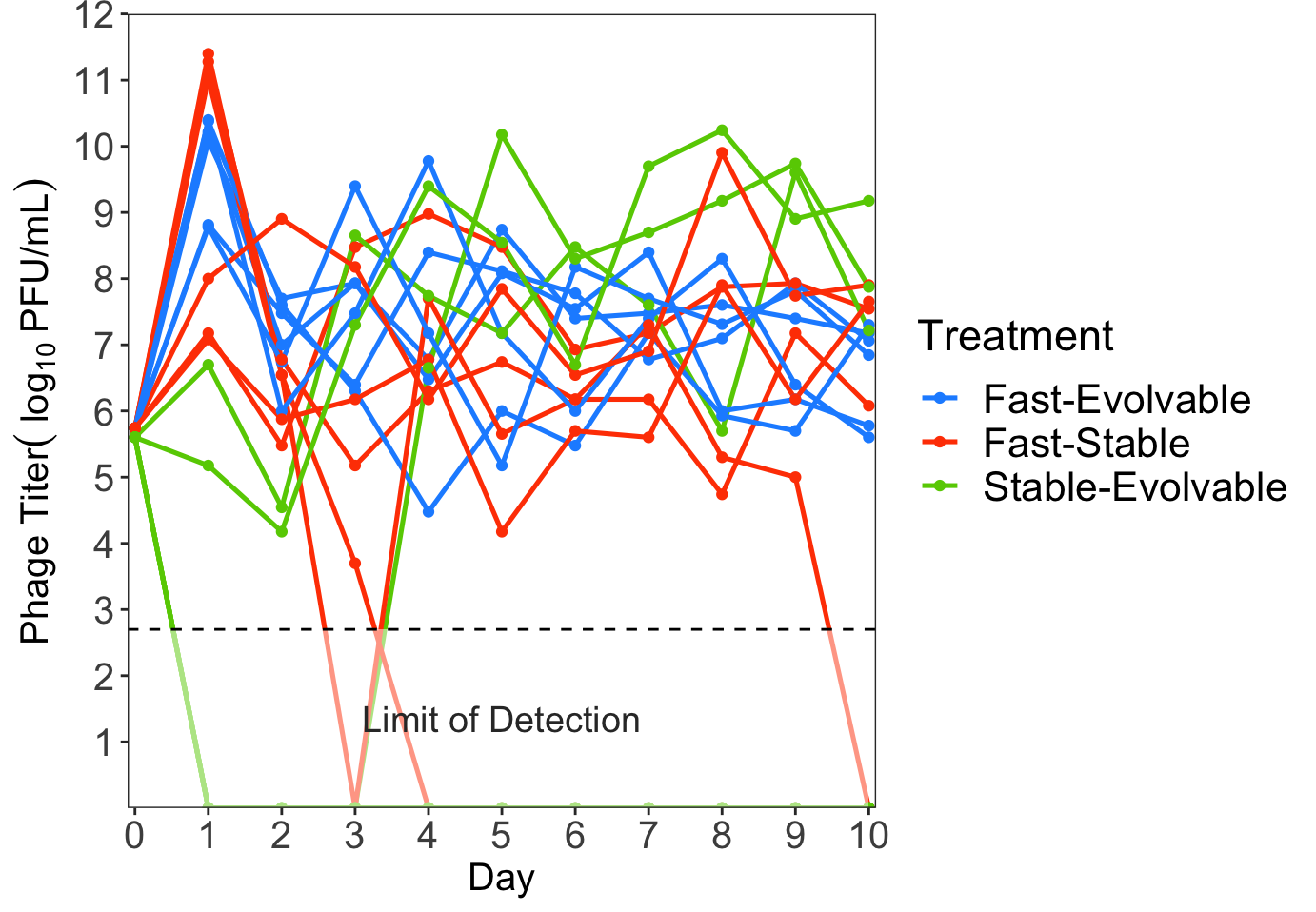


Figure S1 - Phage Titer from suppression experiments presented in Figure 2. Phage densities were measured each day for all replicates of all 3 genotypes.


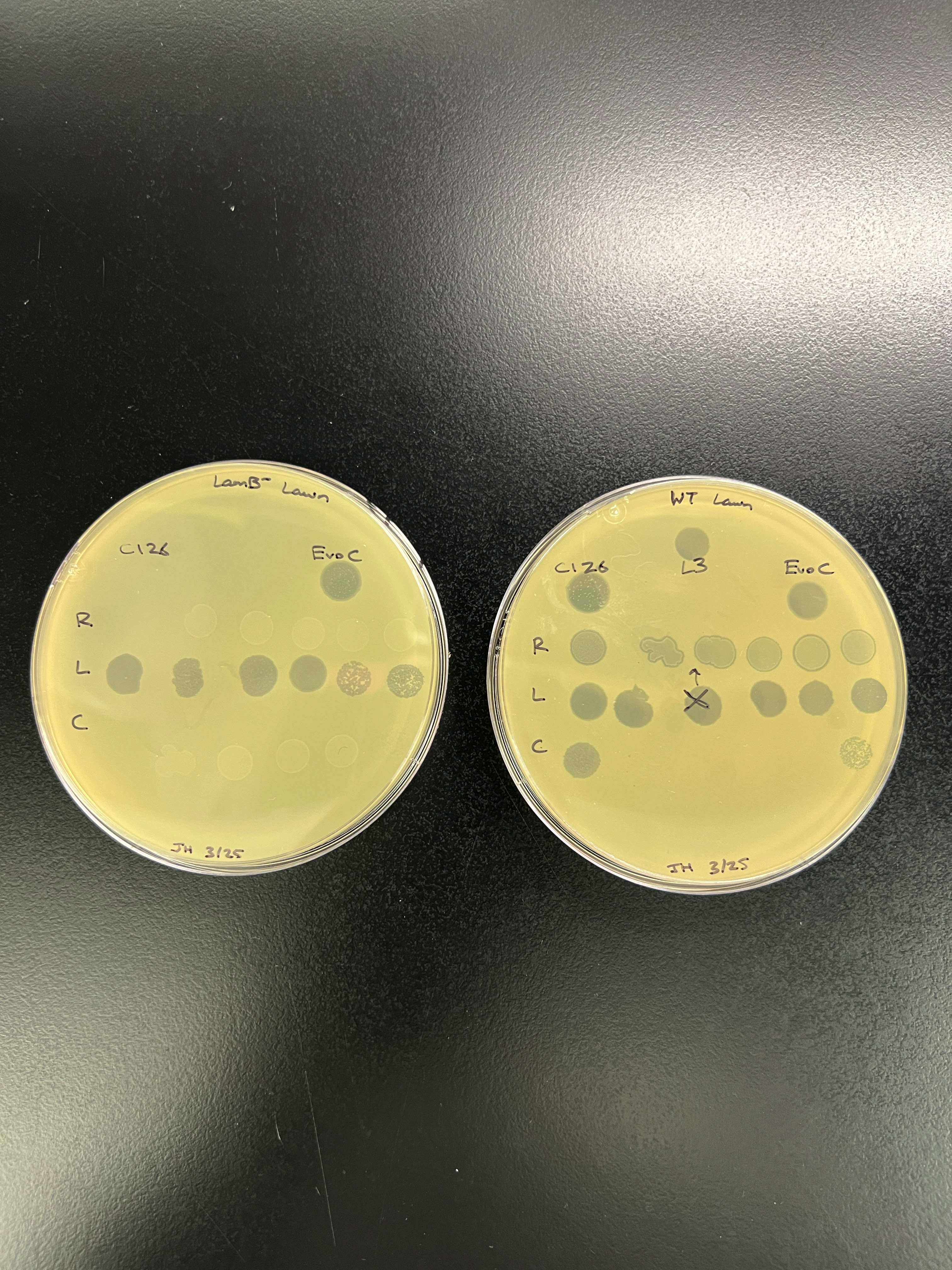


Figure S2 – Clearings on a LamB knockout *E. coli* strain indicating which populations evolved OmpF^+^ phage by day 2. cI26 and EvoC were negative and positive controls, respectively. ‘R’ marks a row of six spots that lack clearing after being spotted with phages from fast-stable populations. ‘L’ indicates a row of clearings for populations spotted from the fast-evolvable populations. ‘C’ indicates a row of spots that lack clearing from stable-evolvable populations.


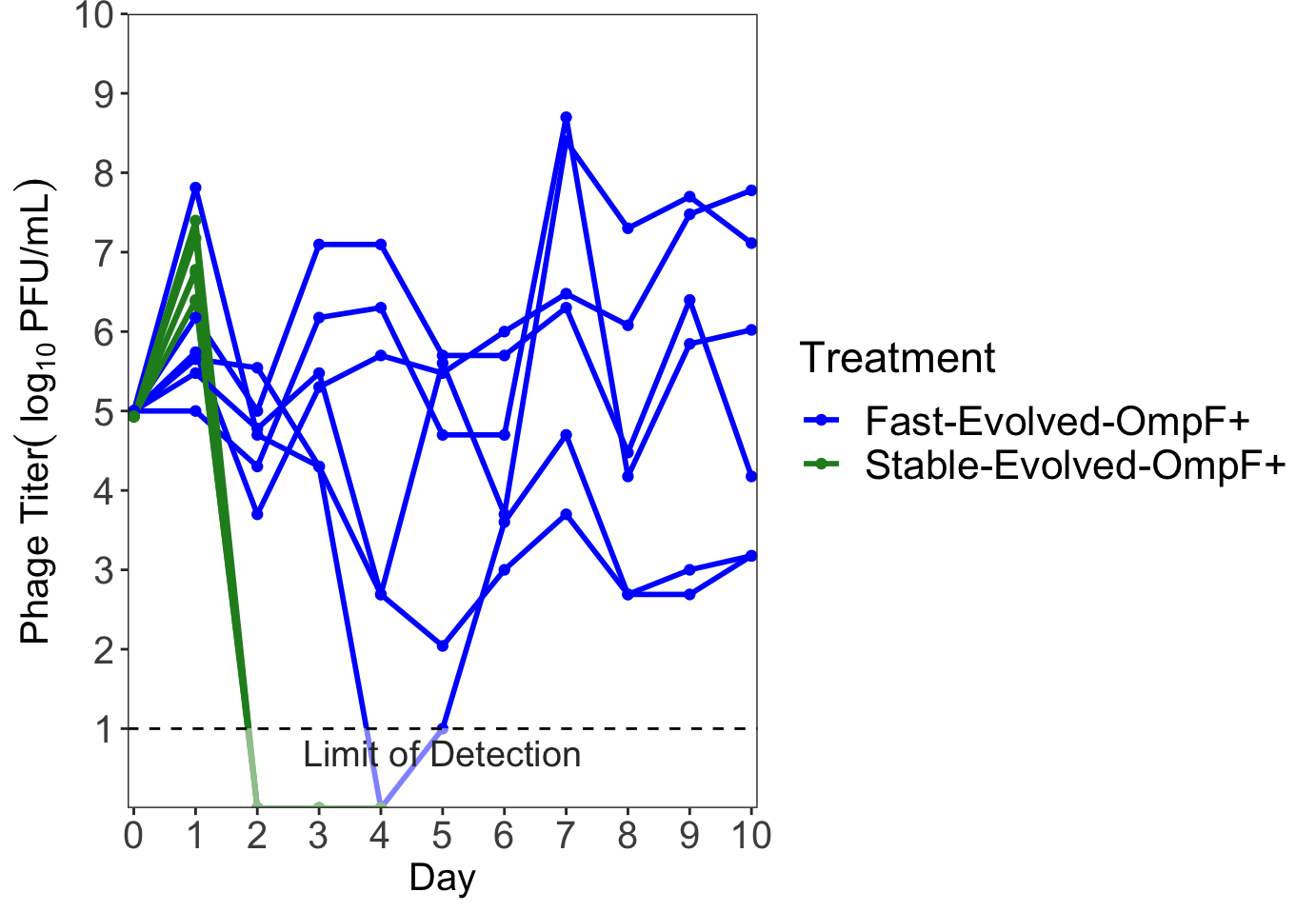


Figure S3 - Phage Titer from suppression experiments presented in Figure 4. Phage densities were measured each day for all replicates of both genotypes.

Figure S4 - Net reproductive rate (reproductive rate + decay rate) of the fast reproducing, unstable and fast reproducing, stable genotypes without the final mutation (“evolvable”) and after receiving the final mutation via engineering (“evolved”). These genotypes were used in the suppression experiment from Figure 4. The net reproductive rate of fast-evolved was slightly higher than and fast-evolvable (2-sample T-test, n = 3 per genotype; t-stat = -3.523, df = 4, P = 0.0244), whereas the net reproductive rates of stable-evolvable and stable-evolved were indistinguishable. (2-sample T-test, n = 3 per genotype; t-stat = -0.09, df = 4, P = 0.933).


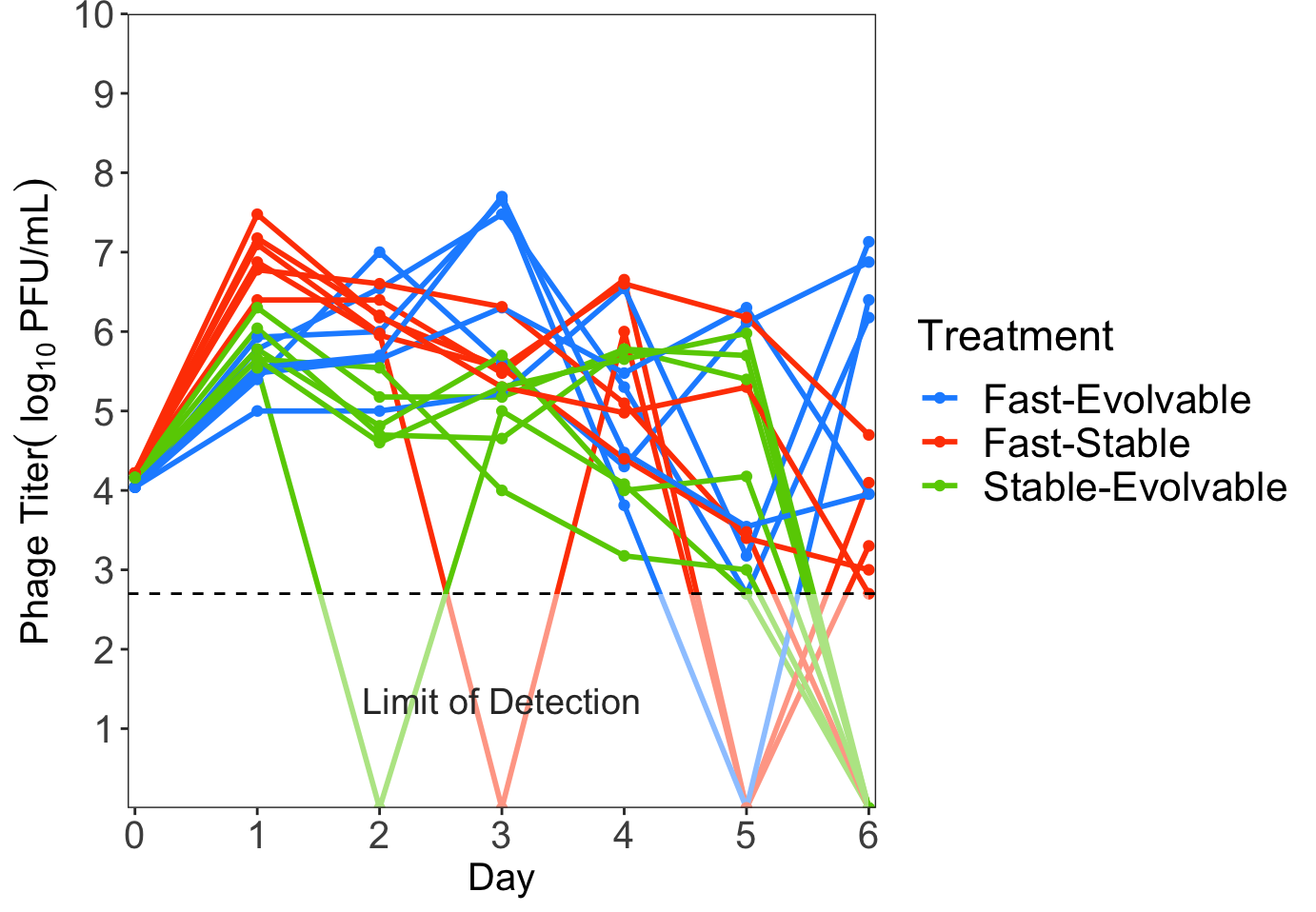


Figure S5 - Phage Titer from suppression experiments presented in Figure 5. Phage densities were measured each day for all replicates of each 3 genotypes.

Table S1 - Phage trait values used to create Figure 1A. Decay rates and evolutionary path length were first reported in Strobel, Horwitz, and Meyer 2022. Reproductive rates were computed from previously reported net reproductive rates and decay rates from the same paper.

| genotype | net reproductive rate per hour | decay rate per hour | reproductive rate per hour | distance to adaptation (# mutations) |
| --- | --- | --- | --- | --- |
| THR 987LEU (Fast-Evolvable) | 1.606601368 | -0.485 | 2.091601368 | 1 |
|  | 1.828305097 | -0.47 | 2.298305097 |  |
|  | 1.714115496 | -0.511 | 2.225115496 |  |
| THR987ARG (Fast-Stable) | 1.631190757 | -0.155 | 1.786190757 | 2 |
|  | 1.873172219 | -0.138 | 2.011172219 |  |
|  | 1.968209044 | -0.119 | 2.087209044 |  |
| THR987CYS (Stable-Evolvable) | 0.798291867 | -0.211 | 1.009291867 | 1 |
|  | 1.041989095 | -0.215 | 1.256989095 |  |
|  | 0.50178022 | -0.22 | 0.72178022 |  |

Table S2 - Phage titers used to initiate suppression experiments.

|  | Suppression Exp. 1 (Figure 2) | Suppression Exp. 2 (Figure 4) | Suppression Exp. 3 (Figure 5) |
| --- | --- | --- | --- |
| Phage Genotype | Phage added to flask replicates | Phage added to flask replicates | Phage added to flask replicates |
| LEU | 5.50E+05 | 1.00E+06 | 1.10E+05 |
| ARG | 5.50E+05 |  | 1.65E+05 |
| CYS | 4.00E+05 | 8.50E+05 | 1.45E+05 |

Table S3 Statistics from Figures 2, 4, and 5

| Figure 2 |  |  |  |
| --- | --- | --- | --- |
| comparison | day | W | P-Value |
| fast-evolvable vs. fast-stable | 1 | 12.5 | 0.4217 |
|  | 2 | 0 | 0.002165 |
|  | 3 | 3.5 | 0.02447 |
|  | 4 | 0 | 0.004847 |
|  | 5 | 1 | 0.004329 |
|  | 6 | 2 | 0.008658 |
|  | 7 | 14 | 0.5887 |
|  | 8 | 12 | 0.3939 |
|  | 9 | 14.5 | 0.6298 |
|  | 10 | 10 | 0.2273 |
| fast-evolvable vs. stable-evolvable | 1 | 5.5 | 0.05382 |
|  | 2 | 0 | 0.004922 |
|  | 3 | 7 | 0.09123 |
|  | 4 | 1 | 0.007687 |
|  | 5 | 5 | 0.04113 |
|  | 6 | 4 | 0.03035 |
|  | 7 | 13 | 0.4848 |
|  | 8 | 10 | 0.2403 |
|  | 9 | 8 | 0.1262 |
|  | 10 | 7 | 0.09155 |
| fast-stable vs. stable-evolvable | 1 | 10 | 0.2281 |
|  | 2 | 21 | 0.6863 |
|  | 3 | 23 | 0.4673 |
|  | 4 | 11 | 0.2928 |
|  | 5 | 28.5 | 0.1087 |
|  | 6 | 26 | 0.2248 |
|  | 7 | 17 | 0.5211 |
|  | 8 | 13.5 | 0.9372 |
|  | 9 | 17 | 0.2281 |
|  | 10 | 10 | 0.7457 |
| Figure 4 |  |  |  |
| comparison | day | W | P-Value |
| fast-evolved vs. stable-evolved | 1 | 2 | 0.01291 |
|  | 2 | 0 | 0.002165 |
|  | 3 | 0 | 0.004998 |
|  | 4 | 13 | 0.468 |
| Figure 5 |  |  |  |
| comparison | day | W | P-Value |
| fast-evolvable vs. fast-stable | 1 | 36 | 0.004922 |
|  | 2 | 21.5 | 0.6304 |
|  | 3 | 9 | 0.1727 |
|  | 4 | 5 | 0.04113 |
|  | 5 | 10 | 0.2403 |
|  | 6 | 0 | 0.004998 |
| fast-evolvable vs. stable-evolvable | 1 | 9 | 0.1712 |
|  | 2 | 10.5 | 0.2615 |
|  | 3 | 8 | 0.132 |
|  | 4 | 6 | 0.06508 |
|  | 5 | 11 | 0.3095 |
|  | 6 | 0 | 0.004998 |
| fast-stable vs. stable-evolvable | 1 | 0 | 0.004998 |
|  | 2 | 10 | 0.2403 |
|  | 3 | 9 | 0.1712 |
|  | 4 | 20.5 | 0.7466 |
|  | 5 | 16.5 | 0.8721 |
|  | 6 | 32 | 0.02919 |
